# Combination of ECT and clozapine in drug-resistant schizophrenia

**DOI:** 10.4103/0019-5545.43067

**Published:** 2005

**Authors:** S. Kurian, P. Tharyan, K.S. Jacob

**Affiliations:** * Reader, Department of Psychiatry, Christian Medical College, Vellore; ** Professor, Department of Psychiatry, Christian Medical College, Vellore; *** Professor, Department of Psychiatry, Christian Medical College, Vellore

Sir,

We read the article by Kumar *et al.*^1^ with interest. The authors have described the development of delirium in a 56-year-old patient who was treated with a combination of electroconvulsive therapy (ECT) and clozapine, and advised caution in the use of this treatment combination. We discuss this issue based on a review of the literature and our clinical experience.

We used a combination of clozapine and ECT in 3 men (21, 24 and 33 years of age) with schizophrenia resistant to treatment with conventional and atypical antipsychotic medications. They were on 350–450 mg of clozapine per day. They received a course of 8–10 ECTs each. They did not have significant cognitive deficits or delirium and completed their courses of ECT. All the 3 patients showed a 40%–50% reduction in their Brief Psychiatric Rating Scale (BPRS) scores.

The literature also supports the use of a combination of ECT and clozapine in those with drug-resistant schizophrenia. Williams *et al.*^2^ have suggested the use of the term ‘neuroleptic-resistant schizophrenia’ as a more positive alternative to the term ‘treatment-resistant schizophrenia’ and have also examined the treatment options for patients resistant to clozapine mono-therapy. Combination with ECT is quoted as a safe and clinically beneficial strategy. Other reports, barring initial concerns about the risk of spontaneous or prolonged seizures with this combination,^3^ also describe the combination of ECT and clozapine to be safe and clinically effective.^4^–^8^ Combining ECT and clozapine has also been advocated for achieving rapid control of disturbed behaviour, when time does not allow for dose titration with clozapine as monotherapy.^9^

Delirium and cognitive deficits during ECT are caused by several factors. In the study by Kumar *et al*., older age, the number and frequency of ECTs, the type and strength of current used, the position of the electrodes or the concomitant use of medication could have resulted in the delirium. Though current guidelines based on case reports do not proscribe the use of ECT with clozapine,^10^ definite guidelines on the safety and efficacy of this combination will have to await the results of the ongoing National Institute of Mental Health (NIMH)-sponsored, multicentre, randomized, open-label efficacy trial comparing ECT augmentation of clozapine versus clozapine monotherapy.^11^ While the combination of ECT and clozapine demands caution, this approach should not be denied to patients with clozapine-resistant schizophrenia.
